# Merkel Cell Carcinoma: Evaluation of the Clinico-Pathological Characteristics, Treatment Strategies and Prognostic Factors in a Monocentric Retrospective Series (n=143)

**DOI:** 10.3389/fonc.2021.737842

**Published:** 2021-12-17

**Authors:** Marco Rastrelli, Paolo Del Fiore, Irene Russo, Jacopo Tartaglia, Alessandro Dal Monico, Rocco Cappellesso, Lorenzo Nicolè, Luisa Piccin, Alessio Fabozzi, Bernardo Biffoli, Claudia Di Prata, Beatrice Ferrazzi, Luigi Dall’Olmo, Antonella Vecchiato, Romina Spina, Francesco Russano, Elisabetta Bezzon, Sara Cingarlini, Renzo Mazzarotto, Alessandro Parisi, Giovanni Scarzello, Jacopo Pigozzo, Tito Brambullo, Saveria Tropea, Vincenzo Vindigni, Franco Bassetto, Daniele Bertin, Michele Gregianin, Angelo Paolo Dei Tos, Francesco Cavallin, Mauro Alaibac, Vanna Chiarion-Sileni, Simone Mocellin

**Affiliations:** ^1^ Soft-Tissue, Peritoneum and Melanoma Surgical Oncology Unit, Veneto Institute of Oncology (IOV)-IRCCS, Padua, Italy; ^2^ Department of Surgery, Oncology and Gastroenterology (DISCOG), University of Padua, Padua, Italy; ^3^ Division of Dermatology, Department of Medicine (DIMED), University of Padua, Padua, Italy; ^4^ Pathological Anatomy Unit, University Hospital of Padua, Padua, Italy; ^5^ Department of Medicine, University of Padua School of Medicine and Surgery, Padua, Italy; ^6^ Unit of Surgical Pathology & Cytopathology, Ospedale dell’Angelo, Mestre, Italy; ^7^ Melanoma Oncology Unit, Veneto Institute of Oncology (IOV)-IRCCS, Padua, Italy; ^8^ Oncology Unit 3, Veneto Institute of Oncology (IOV)-IRCCS, Padua, Italy; ^9^ Clinic of Plastic Surgery, Department of Neuroscience, Padua University Hospital, University of Padua, Padua, Italy; ^10^ Postgraduate School of Occupational Medicine, University of Verona, Verona, Italy; ^11^ Radiology Unit, Department of Imaging and Medical Physics, Istituto Oncologico Veneto (IOV) IRCSS, Padua, Italy; ^12^ Oncology Section, Department of Oncology, Verona University and Hospital Trust, Verona, Italy; ^13^ Department of Radiotherapy, Ospedale Civile Maggiore, Azienda Ospedaliera Universitaria Integrata Verona, Verona, Italy; ^14^ Radiotherapy Unit, Veneto Institute of Oncology, Istituto Oncologico Veneto (IOV)-IRCCS, Padua, Italy; ^15^ Radiotherapy and Nuclear Medicine Unit, Oncological Institute of Veneto IOV-IRCCS, Padua, Italy; ^16^ Department of Medicine (DIMED), Surgical Pathology Unit, University of Padua, Padua, Italy; ^17^ Independent Statistician, Solagna, Italy

**Keywords:** Merkel cell cancer, Merkel carcinoma, Merkel treatment strategies, non-melanoma skin cancer (NMSC), skin cancer

## Abstract

**Background:**

Merkel cell carcinoma (MCC) is a rare neuroendocrine tumor of the skin. The incidence of the disease has undergone a significant increase in recent years, which is caused by an increase in the average age of the population and in the use of immunosuppressive therapies. MCC is an aggressive pathology, which metastasizes early to the lymph nodes. These characteristics impose an accurate diagnostic analysis of the regional lymph node district with radiography, clinical examination and sentinel node biopsy. In recent years, there has been a breakthrough in the treatment of the advanced pathology thanks to the introduction of monoclonal antibodies acting on the PD-1/PD-L1 axis. This study aimed to describe the clinico-pathological characteristics, treatment strategies and prognostic factors of MCC.

**Methods:**

A retrospective cohort study was conducted involving 143 consecutive patients who were diagnosed and/or treated for MCC. These patients were referred to the Veneto Institute of Oncology IOV-IRCCS and to the University Hospital of Padua (a third-level center) in the period between December 1991 and January 2020. In the majority of cases, diagnosis took place at the IOV. However, some patients were diagnosed elsewhere and subsequently referred to the IOV for a review of the diagnosis or to begin specific therapeutic regimens.

**Results:**

143 patients, with an average age of 71 years, were affected mainly with autoimmune and neoplastic comorbidities. Our analysis has shown that age, autoimmune comorbidities and the use of therapy with immunomodulating drugs (which include corticosteroids, statins and beta-blockers) are associated with a negative prognosis. In this sense, male sex is also a negative prognostic factor.

**Conclusions:**

Autoimmune and neoplastic comorbidities were frequent in the studied population. The use of drugs with immunomodulatory effects was also found to be a common feature of the population under examination. The use of this type of medication is considered a negative prognostic factor. The relevance of a multidisciplinary approach to the patient with MCC is confirmed, with the aim of assessing the risks and benefits related to the use of immunomodulating therapy in the individual patient.

## Introduction

Merkel cell carcinoma (MCC) is a rare and highly aggressive malignancy of the skin (1–4). MCC usually presents itself as a rapidly growing pink to red-violet indurated plaque or nodule on sun-damaged skin, most commonly on the head and neck and less frequently on the trunk and extremities (5, 6). Lesions are often asymptomatic and ulceration is uncommon (5, 6). Male predominance is reported and the median age at diagnosis is 75-80 years (7, 8). Risk factors include older age, fair skin, ultraviolet (UV) exposure, immunosuppression, previous malignancies and Merkel Cell Polyomavirus (MCPyV) infection (9). The acronym AEIOU has been coined to encapsulate the main clinical features associated with MCC: asymptomatic, expanding rapidly, immunosuppressed, older than age 50 and UV-exposed (10).

The diagnosis of MCC is based on histopathological and immunohistochemical findings. Histologically, MCC is characterized by dermal and/or subcutaneous nodules or sheets of small, undifferentiated, round-to-oval cells with a vesicular nucleus and scanty cytoplasm (11). The characteristic immunohistological profile demonstrates positive staining with cytokeratins, notably AE1/AE3, CAM5.2, and CK20, and neuroendocrine markers such as chromogranin, synaptophysin, CD56, and NSE (9).

MCC is characterized by a high rate of local recurrence and nodal metastasis, a high mortality rate, and a deep psychological impact (12). The treatment regimen depends on the stage of the disease and includes surgery, radiation, chemotherapy, and/or immunotherapy (4, 7, 13). Surgical treatment consists of a wide excision of the primary lesion, sentinel lymph node biopsy, and/or regional lymph node dissection. Adjuvant radiotherapy may be offered after surgery. Metastatic or inoperable disease could be managed with chemotherapy and/or immunotherapy (4, 13).

There is considerable evidence suggesting that the dysfunction of the immune system contributes significantly to disease progression. This implies that therapies acting on the immune system can prove to be effective in slowing down disease progression (14). Overexpression of PD-L1 is observed in many tumors, including MCC, and allows the tumor to escape immune surveillance which normally enables the immune system to recognize and eliminate any abnormal cell (15). Therefore, the blockage of the interaction between PD-1 and its ligand allows the reactivation of T cells and an effective recruitment of the adaptive immune response. Some monoclonal antibodies capable of acting on this axis are avelumab (anti-PD-L1) and pembrolizumab (anti-PD-1). Both drugs have shown significant clinical efficacy in patients with stage IV MCC and, therefore, they are used as first-line treatment in this subpopulation of patients (16, 17).

Although significant progress has been made in understanding the molecular mechanisms underlying the development of this neoplasm, the characterization of the prognostic factors still remains limited. The importance of this aspect is enhanced by the high mortality rate of MCC and its deep psychological impact on the patient (18).

This study aims to contribute to current literature on MCC by providing an update on consecutive cases of MCC at our institution. This paper describes the demographic, clinical, and diagnostic characteristics of MCC and the therapeutic approach that had a significant prognostic impact.

## Material and Methods

### Study Design

A retrospective cohort study was conducted involving 143 consecutive patients who were diagnosed and/or treated for MCC. These patients were referred to the Veneto Institute of Oncology IOV-IRCCS and to the University Hospital of Padua (a tertiary care facility) in the period between December 1991 and January 2020. In the majority of cases, diagnosis took place at the IOV. However, some patients were diagnosed elsewhere and subsequently referred to the IOV for a review of the diagnosis or to begin specific therapeutic regimens.

### Diagnosis and Treatment

All diagnoses were based on the histopathologic and immunohistochemical examination of the primary tumor. The stage of the disease was determined using the indicators provided by the Eighth edition of the AJCC staging system (19). Performed surgical treatments include wide excision (WE), a treatment performed on the primary lesion; sentinel lymph node biopsy (SNB), typically performed at the same time as the excision of the primary lesion; dissection of the lymph node basin, draining the region of the primary lesion (CLND).

CLND was performed on patients who reported a positive outcome to SNB and on subjects with clinically or radiologically evident lymph node involvement. SNB was performed routinely on all patients with a negative clinical examination of the regional lymph nodes. SNB was omitted in patients whose performance status was so compromised that adequate surgical treatment could not be performed.

The decision on whether to perform radiotherapy and/or chemotherapy was based on specific information concerning individual patient characteristics. The predominant factor influencing this decision was the stage of the disease based on the AJCC system. Possible radiotherapy settings were as follows: adjuvant, in patients who had already undergone surgical treatment for the lesion and/or CLND; neoadjuvant, before carrying out the surgical treatment; palliative, in the case of distant dissemination.

Conventional chemotherapy was reserved for stage IV patients classified in accordance with the AJCC system. Immunotherapy with monoclonal antibodies was also reserved for patients with metastatic disease: to date, this treatment is used as first-line treatment for stage IV patients. Some patients were treated with conventional chemotherapy for first-line treatment and only subsequently referred for immunotherapy.

Patients were subjected to a stringent follow-up regimen for the detection of early disease progression or relapse. Patients were typically seen once every six months for the first five years, then once every twelve months.

Any disease progression was recorded in the database. Disease progression was defined as the onset of distant, lymph node and in-transit metastases or local disease recurrence.

### Data Collection

All data were retrieved from a local prospectively maintained database and entered in a dedicated data sheet for final checks and data analysis.

Study data included demographics, tumor characteristics, comorbidity information including autoimmune and neoplastic comorbidities, and the Charlson Comorbidity Index (20), details on treatment (WE, SNB, CLND, radiotherapy, chemotherapy, and immunotherapy) and prognosis.

Particular attention was paid to the tumor’s immuno-histochemical characteristics, as immunohistochemical analysis was performed for most patients. Neuroendocrine and epithelial markers (such as CK20, NSE, Synaptophysin, Chromogranin, AE1/AE3, MNF 116, and Cam 5.2) were included in the database.

Overall survival was calculated from the date of diagnosis to the date of death, or the patient’s last available visit. Disease-specific survival was calculated from the date of diagnosis to the date of death caused by MCC, or to the date of the last available visit/death not caused by the disease. Finally, disease-free survival was calculated in patients with primitive MCC from the date of diagnosis to the date on which the first relapse arose, or to the date of the last available visit/death.

### Statistical Analysis

Categorical data were summarized as n (%), and compared using the Chi Square test and Fisher’s exact test. Continuous data were reported as medians and interquartile ranges (IQR), and compared using the Mann-Whitney test. Survival curves were estimated using the Kaplan-Meier method and compared by means of the log-rank test. The association between clinically relevant variables and survival was evaluated using Cox regression models and reported as a Hazard Ratio (HR) with a 95% confidence interval (95% CI). The association between survival and chemotherapy was not considered, because chemotherapy was reserved for patients with metastatic disease and was thus a proxy of severe disease rather than a risk factor associated with reduced survival. The limited sample size did not allow any meaningful multivariable analyses. All tests were two-sided and a p-value below 0.05 was considered statistically significant. Statistical analysis was performed using R 4.0 (R Foundation for Statistical Computing, Vienna, Austria) (21).

## Results

### Patients

The analysis included 143 patients (median age at diagnosis 71 years; 74 males and 69 females). Patient characteristics are outlined in **Table 1**. Most of the patients examined presented a primary lesion (110 patients, 77%), while 13 patients presented non-primary lesions (9 metastatic patients, and 4 disease recurrences). With regard to the initial clinical staging, 48% of patients were stage III (the most commonly attributed stage), 27% stage I, 15% stage II, and 10% stage IV. Limbs were the most common location of the lesion (57%), followed by the head/neck area (23%), and the trunk/buttocks (20%). Immunohistochemical analysis of the bioptic material was performed in 99 patients (69.23% of the total). The most commonly detected immunohistochemical markers were cytokeratin 20 (in 75% of lesions), synaptophysin (83%), NSE (26%), chromogranin (69%), AE1/AE3 (17%), MNF 116 (32%), and CAM 5.2 (33%). The male sex was associated with worse disease-free survival. Neoplastic comorbidities were found in 24% of patients and autoimmune comorbidities in 27% (the most frequently encountered were Type 1 diabetes mellitus, rheumatoid, or psoriatic arthritis). Specifically, 6% of patients were affected by hematological neoplasms (mainly Non-Hodgkin’s Lymphoma, chronic lymphocytic Leukemia, and Myeloproliferative or Myelodysplastic syndromes). Many of the patients examined were on immunomodulatory medications (59 patients, 41%), while 23 patients (16%) were using statins. The most commonly used immunomodulatory drugs were corticosteroids (16 patients, 11%) and beta-blockers (23 patients, 16%).

**Table 1:** Characteristics of 143 patients who had a diagnosis of MCC between December 1991 and January 2020.

		All patients	Non-primary tumors	Primary tumors
	**N patients**	143	33	110
**Demographics**	Age at diagnosis, years^a^	71 (63-79)	71 (63-76)	71 (62-79)
	Sex:			
	Female	69 (55)	13 (19)	56 (81)
	Male	64 (45)	20 (27)	54 (73)
	Family history of cancer:			
	No	37 (26)	7 (21)	30 (27)
	Yes	15 (10)	4 (12)	11 (10)
	Information not available	91 (64)	22 (67)	69 (63)
**Merkel cell Carcinoma**	Presentation:			
	Primary	110 (77)	0 (0)	110 (100)
	Occult primary	24 (17)	24 (73)	0 (0)
	Metastatic	5 (3)	5 (15)	0 (0)
	Recurrence	4 (3)	4 (12)	0 (0)
	Tumor size:			
	≤2 cm	38 (27)	0	38 (35)
	>2 cm	105 (73)	33 (100)	72 (65)
	Anatomic location:			
	Head/neck	32 (23)	1 (3)	31 (28)
	Extremities	82 (57)	11 (33)	71 (65)
	Trunk/buttocks	29 (20)	21 (64)	8 (7)
	Tumor stage:			
	I	38 (27)	0 (0)	38 (35)
	II	22 (15)	1 (3)	21 (19)
	III	68 (48)	25 (76)	43 (39)
	IV	15 (10)	7 (21)	8 (7)
**Comorbidity**	Age-adjusted Charlson comorbidity index^a^	4 (2-5)	3 (2-4)	4 (2-5)
	Neoplastic comorbidity:			
	No	109 (76)	28 (85)	81 (74)
	Yes	34 (24)	5 (15)	29 (16)
	Autoimmune comorbidity:			
	No	104 (73)	22 (67)	82 (75)
	Organ-specific	14 (10)	3 (9)	11 (10)
	Systemic	12 (8)	4 (12)	8 (7)
	Both	13 (9)	4 (12)	9 (8)
**Drugs**	Immunomodulatory:			
	No	84 (59)	20 (61)	64 (58)
	Yes	59 (41)	13 (39)	46 (42)
	Statins:			
	No	120 (84)	27 (82)	93 (85)
	Yes	23 (16)	6 (18)	17 (15)
**Immunohistochemistry**	Immunohistochemistry availability, N patients	99	21	78
	CK20: expression	74/99 (75)	17/21 (81)	57/78 (73)
	NSE: expression	26/99 (26)	3/21 (14)	23/78 (29)
	Synaptophysin: expression	82/99 (83)	18/21 (86)	64/78 (82)
	Chromogranin: expression	68/99 (69)	14/21 (67)	54/78 (69)
	AE1 AE3: expression	17/99 (17)	4/21 (19)	13/78 (17)
	MNF 116: expression	32/99 (32)	6/21 (29)	26/78 (33)
	CAM 5.2: expression	33/99 (33)	8/21 (38)	25/78 (32)

Data expressed as n (%) or ^a^median (IQR).

### Treatment

**Figure 1:**
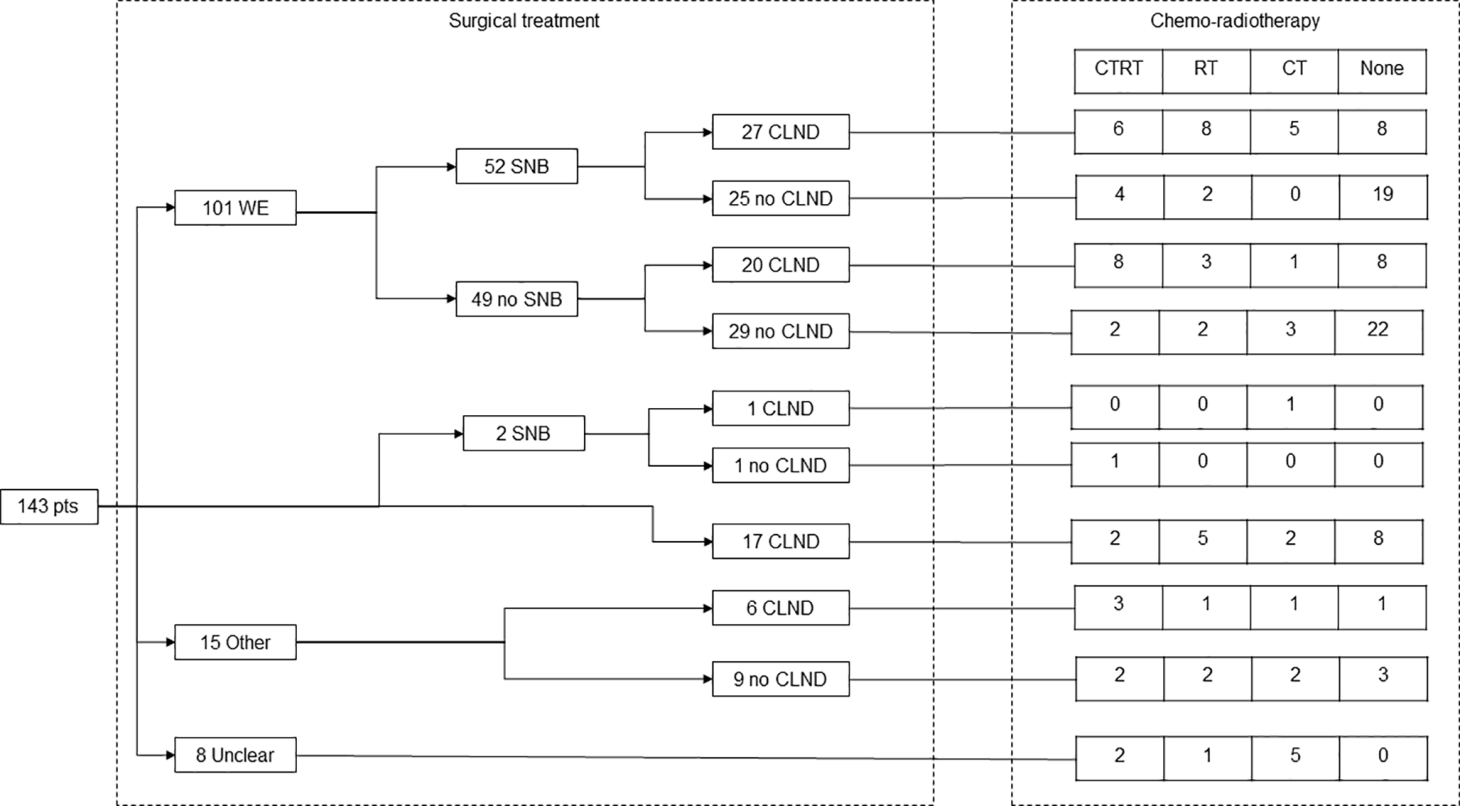
Flow-chart of treatment strategy in 143 patients who had a diagnosis of MCC between December 1991 and January 2020.

**Figure 1** summarizes the treatment strategy for MCC patients in this study.

Wide excision (WE) was the most common treatment for the primary lesion (101 patients, 71%). Of these, 52 patients also underwent SNB. Two patients underwent direct sentinel lymph node biopsy and 17 CLND. Fifteen patients underwent other treatments (such as wide resection and locoregional perfusion of the limb), while 8 patients were treated at other centers and it was not possible to retrieve their surgical details.

Following SNB, CLND identified a median of 1 positive lymph node (IQR 0-6). CLND was also performed in 5 patients with negative SNLB (median 3 positive lymph nodes, IQR 0-5).

Radiotherapy was administered to 54 patients (in 35 of the patients who received radiotherapy, the intent was adjuvant) and chemotherapy to 50 patients. Of these, 17 patients were treated using monoclonal antibodies acting on the PD-1/PD-L1 axis (avelumab or pembrolizumab).

### Survival

**Figure 2:**
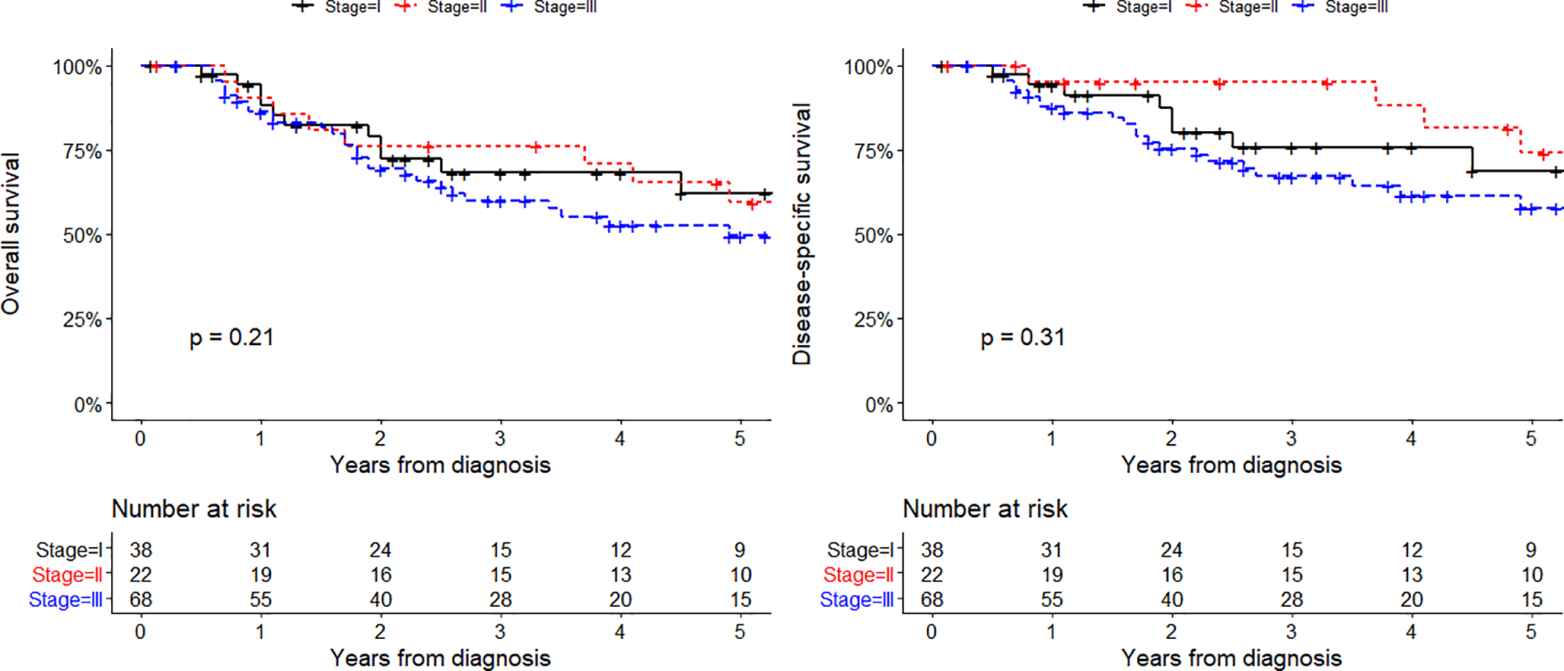
Overall survival (left) and disease-specific survival (right) in 128 patients who had a diagnosis of stage I-III MCC between December 1991 and January 2020.

The median follow-up in 128 stage I-III patients was 31 months (IQR 15-62). Thirty-seven patients died from the disease and 19 patients died from other causes. The five-year overall survival rate was 62-59-50% in patients with stage I-II-III (p = 0.21). The five-year disease-specific survival rate was 69-74-58% in patients with stage I-II-III (p = 0.31). (**Figure 2**). Univariate analyses of overall survival and disease-specific survival are shown in **Table 2**. Impaired overall survival was associated with older age (HR 1.03, 95% CI 1.00-1.06) and a higher Charlson Comorbidity Index (HR 1.16, 95% CI 1.01-1.33). Impaired disease-specific survival was associated with the presence of autoimmune comorbidities (HR 2.00, 95% CI 1.03-3.91) and the use of immunomodulatory drugs (HR 2.94, 95% CI 1.52-5.67).

**Table 2:** Univariate analysis of overall survival and disease-specific survival in 128 patients who had a diagnosis of stage I-III MCC between December 1991 and January 2020.

	Overall survival		Disease-specific survival	
	HR (95% CI)	p-value	HR (95% CI)	p-value
Primary vs. non primary tumor	1.02 (0.54 to 1.90)	0.96	1.05 (0.50 to 2.30)	0.90
Age at diagnosis	1.03 (1.00 to 1.06)	0.04	1.00 (0.97 to 1.03)	0.90
Male vs. female	1.69 (0.98 to 2.91)	0.06	2.24 (1.14 to 4.41)	**0.02**
Anatomic location:				
Head/neck vs. extremities	1.15 (0.59 to 2.22)	0.67	1.62 (0.74 to 3.56)	0.23
Trunk/buttocks vs. extremities	1.35 (0.70 to 2.59)	0.37	1.81 (0.83 to 3.97)	0.14
Tumor size: >2 cm vs. ≤2 cm	1.57 (0.81 to 3.05)	0.18	1.42 (0.64 to 3.10)	0.39
Tumor stage: III vs. I-II	1.61 (0.93 to 2.77)	0.09	1.67 (0.86 to 3.25)	0.13
Age-adjusted Charlson comorbidity index	1.16 (1.01 to 1.33)	0.03	1.12 (0.94 to 1.33)	0.21
Neoplastic comorbidity: yes vs. no	0.93 (0.49 to 1.76)	0.82	1.13 (0.53 to 2.39)	0.76
Autoimmune comorbidity: yes vs. no	1.43 (0.79 to 2.59)	0.23	2.00 (1.03 to 3.91)	**0.04**
Immunomodulatory drugs:				
Corticosteroids vs. no drugs	1.46 (0.68 to 3.15)	0.33	2.19 (0.93 to 5.17)	0.07
Beta blockers and statins v. no drugs	1.10 (9.53 to 2.23)	0.79	1.94 (0.89 to 4.26)	0.09
CK20: expression vs. absence	1.46 (0.68 to 3.12)	0.34	1.85 (0.77 to 4.47)	0.17
NSE: expression vs. absence	0.69 (0.31 to 1.52)	0.35	0.75 (0.32 to 1.75)	0.50
Synaptophysin: expression vs. absence	2.02 (0.77 to 5.30)	0.15	1.63 (0.61 to 4.34)	0.33
Chromogranin: expression vs. absence	1.13 (0.54 to 2.35)	0.75	1.72 (0.70 to 4.22)	0.24
AE1 AE3: expression vs. absence	0.43 (0.10 to 1.84)	0.26	0.26 (0.04 to 1.94)	0.19
MNF 116: expression vs. absence	0.96 (0.46 to 2.01)	0.92	0.93 (0.41 to 2.10)	0.86
CAM 5.2: expression vs. absence	1.15 (0.58 to 2.27)	0.69	0.90 (0.42 to 1.93)	0.78
Radiotherapy: yes vs. no	1.46 (0.85 to 2.51)	0.17	2.01 (1.07 to 3.99)	**0.03**

The bold values are statistically significant.

SNB was found to be positive in 19 patients and negative in 24 patients who received SNB concurrently with WE. Patients with positive SNB had worse overall survival rate (HR 4.44, 95% CI 1.15-17.16; p = 0.03) and disease-specific survival (HR 3.96, 95% CI 1.00-15.72; p = 0.04) with respect to patients with negative SNB. In the same subgroup, having 3 or more positive lymph nodes at CLND was not associated with a worse overall survival rate (HR 1.77, 95% CI 0.68-4.61; p = 0.24) or disease-specific survival (HR 3.09, 95% CI 0.97-9.88; p = 0.06) when compared to patients with 2 or less positive lymph nodes.

**Figure 3:**
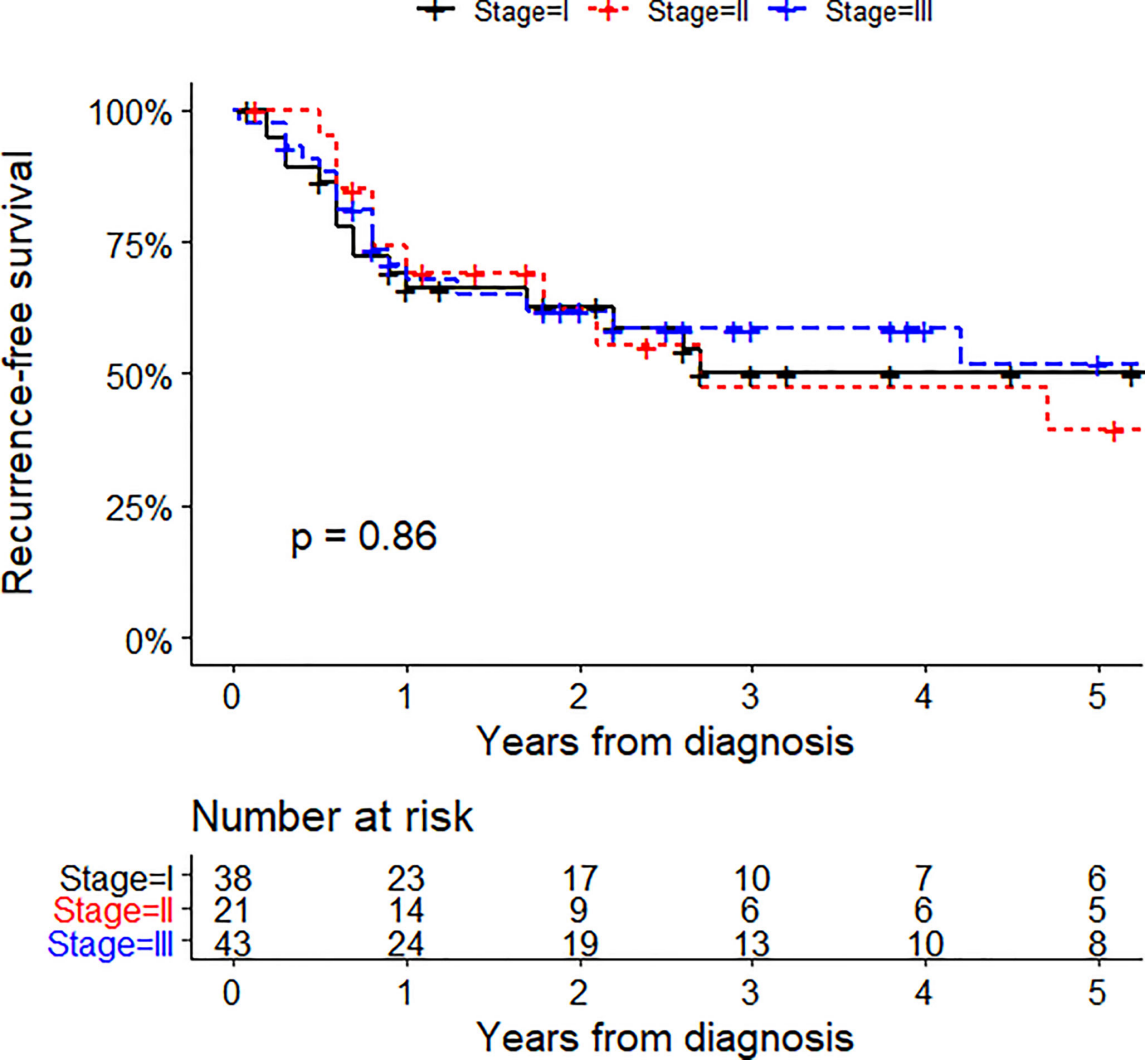
Recurrence-free survival in 102 patients who had a diagnosis of stage I-III primary MCC between December 1991 and January 2020.

At the time of the analysis, 43 of the 102 stage I-III patients with primary disease relapsed (43%). Local recurrence was observed in 11 patients, in-transit metastases in 4 patients, lymph node metastases in 15 patients, and distant metastases in 13 patients. The five-year recurrence-free survival rate was 43% (**Figure 3**). A univariate analysis of recurrence-free survival is shown in **Table 3**. Impaired disease-free survival was associated with receiving immunomodulatory drugs (HR 2.51, 95% CI 1.36-4.57; p = 0.003) and radiotherapy (HR 2.74, 95% CI 1.50-5.03; p = 0.001). Among stage I-III patients with primary disease who received SNB concurrently with WE, recurrence-free survival was not associated with the positivity of SNB (HR 1.00, 95% CI 0.38-2.65; p = 0.99). In the same subgroup, having three or more positive LNs at CLND was not associated with recurrence-free survival (HR 2.05, 95% CI 0.85 to 4.94; p = 0.11) with respect to having two or less positive LNs at CLND.

**Table 3:** Factors associated with recurrence-free survival among patients with primary stage I-III MCC.

	Recurrence-free survival	
	HR (95% CI)	p-value
Age at diagnosis	1.01 (0.98 to 1.03)	0.58
Male vs. female	1.33 (0.74 to 2.42)	0.34
Anatomic location:		
Head/neck vs. extremities	0.85 (0.42 to 1.70)	0.64
Trunk/buttocks vs. extremities	1.07 (0.93 to 3.07)	0.90
Tumor size: >2 cm vs. ≤2 cm	0.98 (0.53 to 1.82)	0.95
Tumor stage III vs. I-II	0.86 (0.47 to 1.59)	0.65
Age-adjusted Charlson comorbidity index	1.09 (0.92 to 1.27)	0.35
Neoplastic comorbidity: yes vs. no	1.07 (0.53 to 2.10)	0.87
Autoimmune comorbidity: yes vs. no	1.06 (0.98 to 1.14)	0.17
Immunomodulatory drugs:		
Corticosteroids vs. no drugs	1.02 (0.39 to 2.65)	0.97
Beta blockers and statins v. no drugs	2.53 (1.27 to 5.05)	**0.008**
CK20: expression vs. absence	1.16 (0.53 to 2.57)	0.71
NSE: expression vs. absence	0.75 (0.34 to 1.63)	0.47
Synaptophysin: expression vs. absence	1.71 (0.65 to 4.51)	0.28
Chromogranin: expression vs. absence	1.19 (0.55 to 2.58)	0.66
AE1 AE3: expression vs. absence	0.60 (0.18 to 1.99)	0.41
MNF 116: expression vs. absence	0.94 (0.45 to 2.00)	0.88
CAM 5.2: expression vs. absence	0.62 (0.28 to 1.38)	0.24
Radiotherapy: yes vs. no	2.74 (1.50 to 5.03)	**0.001**

The bold values are statistically significant.

## Discussion

This study provides an update on MCC consecutive cases treated at our institution, confirming previous findings (18) in a larger sample of patients (143 v. 90). The prognostic features found in this study are explained below.

Therapy performed using immunomodulatory drugs was one of the main factors associated with worsened prognosis. 59 patients (41% of the total) were on immunomodulatory drug therapy, a category that does not only include drugs used in the treatment of autoimmune or inflammatory diseases (such as corticosteroids, azathioprine, or tacrolimus), but also other drugs exerting an effect on the immune system. Among these, we included statins and beta blockers. From the analysis of the literature, emerges the immuno-modulating role of pharmacological agents such as beta-blockers and statins. As for HMG-CoA reductase inhibitors, their non-LDL-c lowering properties could be involved in immunomodulation. This effect could occur both through mevalonate pathway-dependent and independent mechanisms. Then, statins are able to interfering with the expression of MHC molecules and to inducing lymphocyte class switch. These effects could determine an increased incidence of Merkel cell carcinoma in patients who chronically use these drugs. Furthermore, many evidences supporting an immune-modulating role of beta-blocking agents. In fact, adrenaline promotes the activation of the immune system against cancer cells by activating NK cells through signaling of the beta-2 adrenergic receptor. Finally, the beta-blockers could promote the expression of CD107a and HLA-DR on cytotoxic T cells (22–24). We propose these pharmacological effects could determine a state of sub-clinical immunomodulation (a phenomenon distinct from the immunosuppression which is seen, for example, in transplant patients) which, could cause an increased incidence of Merkel cell carcinoma. The use of drugs with immunomodulatory effects was found to be associated with worse disease-specific survival. This association is in line with the data reported in the literature (25). In fact, in this subpopulation of patients, one could hypothesize the presence of an iatrogenic immunosuppression which could justify worsened survival. It is known that immunocompromised patients are characterized by a worse prognosis than immunocompetent patients (26, 27). Based on this observation, it might be advantageous to review the patient’s therapeutic regimen and weigh the potential benefits in order to reduce the extent of iatrogenic immunosuppression and consequently improve the prognosis.

The expression of epithelial and neuroendocrine immunohistochemical markers (CK20, NSE, Synaptophysin, Chromogranin, AE1/AE3, MNF 116, and Cam 5.2) did not appear to be significantly correlated with survival outcome, unlike the previous study where the lack of CK20 expression in immunohistochemical markers was associated with better survival (18).

The presence of a high number of comorbidities (expressed by the Charlson Comorbidity Index) correlates with reduced survival. These data are in line with the already existing international literature (28). The relatively high proportion of MCC patients with hematologic malignancies is consistent with the evidence described by other investigators, who report a percentage of about 5% of patients affected by these comorbidities (29). This association might find a potential explanation in the putative cell of origin of MCC from B-cell precursors (30) and/or the presence of immunological changes (often subclinical) in patients affected by chronic lymphocytic leukemia and other lymphoproliferative disorders (30). Although the origin of MCC cells from pre/pro B-cells appears unlikely, given the lack of experimental evidence regarding the fact that these cells are able to assume a phenotype similar to MCC (31), it is not possible to define with certainty the main factor underlying the described association.

Although the literature analysis reveals the presence of an association between the number of positive lymph nodes following CLND and survival (32, 33), such an association did not emerge in the present study.Data in the literature concerning the association between SNB positivity and survival are discordant (33). The present study showed a clear association between SNB positivity and a worse prognosis. This data testifies to the importance of adequate treatment of the regional lymph nodes (with CLND and/or radiotherapy, often combined) in this subpopulation of patients.

As for comorbidities, patients with autoimmune conditions are characterized by a worse prognosis, probably due to the intake of immunomodulatory drugs (18).

Radiation therapy was linked to reduced survival; however, this association could be influenced by the fact that patients undergoing radiotherapy are characterized by a more advanced stage (18% of patients presented with a clinical stage <II and 72% with a stage> II) of disease (lymph node or distant metastases) (8).

In our study we confirm the data present in the literature relating to Merkel cell tumor, which define the typical age of the patient, the localization of the tumor and the expression of epithelial and neuroendocrine markers.

## Conclusion

Autoimmune and neoplastic comorbidities were frequent in the studied population. The use of drugs with immunomodulatory effects was also found to be a common feature of the population under examination. The use of this type of medication is considered a negative prognostic factor. The relevance of a multidisciplinary approach to the patient with MCC is confirmed, with the aim of assessing the risks and benefits related to the use of immunomodulating therapy in the individual patient.

### Strengths and Weaknesses of the Study

The strengths of this study include the large monocentric sample and the importance attributed to the analysis of comorbidities. This experience has allowed us to validate almost all previous prognostic features and to design a future national collaborative study.

Limitations are related to the lack of data regarding the diagnosis of certain patients and their therapy (missing data about expression of immunohistochemical markers, type and intent of clinical treatment). This study, in fact, took place over a very long period (from 1991 to 2020), which is why several data routinely recorded today (such as the expression of immunohistochemical markers) were not available for patients who were enrolled in the early stages. It is also necessary to consider the diagnostic and therapeutic heterogeneity characterizing such a prolonged period.

## Data Availability Statement

The raw data supporting the conclusions of this article will be made available by the authors, without undue reservation.

## Ethics Statement

The studies involving human participants were reviewed and approved by Ethics Committee of the Veneto Institute of Oncology (Approval No. 0015918 CESC-IOV) on 21 September 2020. The patients/participants provided their written informed consent to participate in this study.

## Author Contributions

Study concepts: PF, IR, MR, JT, RC, and LN. Study design: PF, FC, MR, MA, and SM. Data acquisition: PF, FC, BF, BB, FR, RS, DB, ADM, AF, LP, ST, EB, SC, and RM. Quality control of data and algorithms: PF and FC Data analysis and interpretation: PF, FC, and SM. Statistical analysis: FC. Manuscript preparation: PF, IR, JT, and FC. Manuscript editing: PF, FC, SM, LD, and MR. Manuscript review: SM, MR, VC-S, JP, TB, AF, FB, MG, AD, RC, MA and RM. All authors contributed to the manuscript’s revision, and read and approved the submitted version.

## Conflict of Interest

The authors declare that the research was conducted in the absence of any commercial or financial relationships that could be construed as a potential conflict of interest.

## Publisher’s Note

All claims expressed in this article are solely those of the authors and do not necessarily represent those of their affiliated organizations, or those of the publisher, the editors and the reviewers. Any product that may be evaluated in this article, or claim that may be made by its manufacturer, is not guaranteed or endorsed by the publisher.
